# Overexpression of transcription factor *SlWRKY28* improved the tolerance of *Populus davidiana* × *P. bolleana* to alkaline salt stress

**DOI:** 10.1186/s12863-020-00904-9

**Published:** 2020-09-14

**Authors:** Xin Wang, Zainab Ajab, Chenxi Liu, Songmiao Hu, Jiali Liu, Qingjie Guan

**Affiliations:** Key Laboratory of Saline-alkali Vegetation Ecology Restoration, Northeast Forestry University, Harbin, 150040 China

**Keywords:** *SlWRKY28*, *Salix linearistipularis*, *Populus davidiana* × *P. bolleana*, WRKY transcription factor, Transgenesis, Adversity stress

## Abstract

**Background:**

WRKY transcription factors (TFs) have been suggested to play crucial roles in the response to biotic and abiotic stresses. This study is the first to report the alkaline salt regulation of the WRKY gene.

**Results:**

In this study, we cloned a WRKY gene (*SlWRKY28*) from the *Salix linearistipularis* and then transferred to the *Populus davidiana* × *P. bolleana* for expression. Sequence analysis on the transcriptome of *Salix linearistipular* showed the significant up-regulation of WRKY gene expression in response to salt-alkali stress in seedlings. Our data showed that SlWRKY28 localized to the nucleus. Furthermore, the expression of the *SlWRKY28* from female plants increased with saline-alkali stress according to the northern blot analysis results. The results of 3,3′-Diaminobenzidine (DAB) staining showed that hydrogen peroxide (H_2_O_2_) concentration was lower under stress, but ascorbate peroxidase (APX) enzyme activity was significantly higher in the overexpressed plants than that in non-transgenic (NT) plants.

**Conclusions:**

We found out the *SlWRKY28* induced regulation of the enzyme gene in the reactive oxygen species (ROS) scavenging pathway is a potential mechanism for transgenic lines to improve their resistance to alkaline salt. This study shows theoretical and practical significance in determining *SlWRKY28* transcription factors involved in the regulation of alkaline salt tolerance.

## Background

Plants are constantly challenged by various factors that affect plant growth and development throughout their life cycle. Genetically engineered plants have been developed in a wide range of tree species, and numerous transgenic clones with improved traits have been generated, some of which have undergone field trials to the environmental release stage; the genetic stability of genetically modified forests has been verified in field trials [1]. A saline-alkali meadow steppe has characteristics of sparse vegetation and fragile plant ecosystem. Species richness is a determining factor for the availability of plant resources during vegetation restoration. *Salix linearistipularis* is a unique deciduous shrub growing in saline-alkali meadow steppes [2–4]**.** and can endure a soil habitat of above pH 9.2. *S.linearistipularis* nearly has the same crude protein content as *Melilotus suavcolen* and alfalfa. *Salix linearistipularis*is, an essential woody resource for ecological restoration and afforestation of soda saline-alkali grassland 2. The growth and development of forest trees are frequently challenged by biotic (such as pests and diseases) and abiotic (such as drought, soil salinity, and flooding) stresses, although natural forests have evolved a certain ability to cope with these adverse environmental factors. Sequence analysis of transcriptional sets in *S. linearistipularis* under salt stress showed agreement with 22 pathways regulated by salt stress in *Arabidopsis thaliana* and node genes, including 1397 up-regulated and 1637 down-regulated genes. *SlWRKY* gene is one of the up-regulated genes 4. WRKY protein is a specific transcription factor that can be used to regulate biotic and abiotic stresses [5], including responses to salt stress [6], drought stress [7], cold stress [8], and injury response [9]. There are 74 members of the WRKY protein family for *A. thaliana* [10], 102 members for rice [11], 136 members for corn [12], and 104 members for poplar [13]. At present, the stress responses of plants with WRKY proteins have been widely reported [14]. WRKY protein binding reaction element W-boxes [(T)(T)TGAC(C/T)] are located in the upstream regulatory region of defense-related gene promoters [15]. Therefore, the WRKY protein may regulate downstream gene expression related to stress response and potentially improve resistance through metabolic component regulation. The expression of *BCWRKY32* and *BCWRKY35* genes in the adventitious roots of *Bupleurum* are induced by NaCl [16]. In the transgenic plants of *A. thaliana* overexpressing the AtMBF1 gene, the expression of *AtWRKY18*, *AtWRKY33*, *AtWRKY40*, and *AtWRKY46* increased; heat tolerance was also significantly higher than that of wild type plants [17]. The WRKY family in grapes has 59 members, of which 36 have twofold up-regulated expression under low-temperature stress. The overexpression of the *WRKY43* gene increases the cold tolerance of transgenic plants; thus, the gene is involved in the signal transduction process of cold stress in grapes [18]. The *WRKY* gene of *Tamarix rubrum* is induced by using high salt conditions, drought, and exogenous ABA application. The overexpression of *WRKY4* gene increases the tolerance of transgenic plants to saltation and abscisic acid (ABA) [19]. A WRKY6 can bind to two W-boxes located at the upstream of phosphate transport-related gene PHO1 promoter and negatively regulates AtPHO1 expression. Transgenic plants overexpressing *AtWRKY6* are sensitive to phosphate deficiency and accumulate small amounts of phosphorus in their stems [20, 21] found that *WRKY18, 38, 53, 54, 58, 59, 66,* and *70* genes are directly regulated by NPRI. WRKY TFs have been implicated in the regulation of different metabolic pathways (e.g. biosynthesis of secondary metabolites, plant senescence and signal molecule-delivery) under biotic and abiotic stresses in plants [22]. WRKY TFs are plant-specific proteins and constitute one of the largest TF families in plants. In *A. thaliana*, WRKY TFs can directly activate genes related to salicylic (SA), jasmonic acid (JA), and H_2_O_2_ biosynthesis and improve stress tolerance by participating in the metabolic regulation of ROS stress [23–25]*.* A single WRKY TF can respond to multiple stress factors simultaneously and contribute to the regulation of other physiological processes. Currently, the potential roles of the *WRKY* gene in the tolerance of plants to alkaline salt have not been reported.

*Populus davidiana* × *P. bolleana* is an artificial hybrid combination of *Populus davidiana* as the female parent and Populus bolleana Lauche as the male parent, which was further selected and bred [26]. The trunk is straight, the petiole and leaf backs are silver-white downy, the leaf surface is dark green, and the branches and leaves are shaken by the wind, adding to its beauty. The inflorescence falls off nature ally without flying flocculent, which does not cause environmental pollution. *Populus davidiana* × *P. bolleana* has a strong cold wintering ability, but also very drought-resistant, and rapid growth, the tree posture is beautiful and neat, suitable for the northern drought and cold natural conditions, is China’s northern narrow-crowned poplar in the most cold, the most beautiful of the preferred species of landscaping and protection [27]. At present, the *Populus davidiana* × *P. bolleana* tissue culture propagation technology has been quite mature, the tissue culture expansion can get a large number of seedlings, and has the characteristics of rapid growth, beautiful tree shape, has become a good model of research woody plants .

In this study, *SlWRKY28* from *S. linearistipularis* was cloned and comfirmed to be associated with the saline-alkali stress response. The expression of *SlWRKY28* in *Populus davidiana* × *P. bolleana* improved the tolerance to saline-alkali stress.

## Results

### Bioinformatics analysis of *SlWRKY28*

**Fig. 1:**
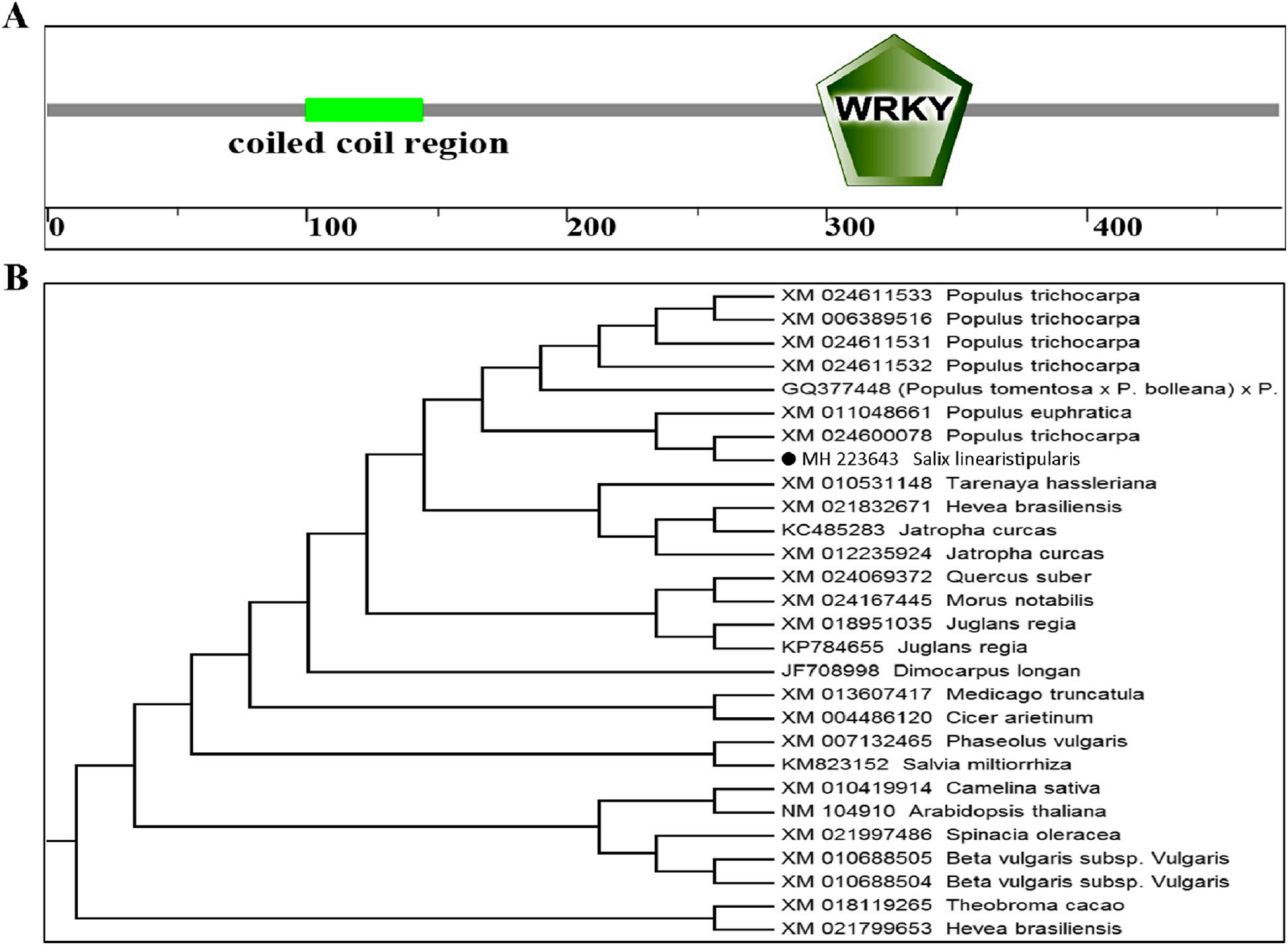
Conserved domains and phylogenetic tree of *SlWRKY28***. a** The conserved retrotransposon gag and WRKY domain were found in the amino acid sequence. WRKYGQK was between 296 and 356 amino acids. **b** Phylogenetic trees based on the amino acid sequence of SlWRKY28 and homologous sequences from the GenBank database. The representative sequences reported in this study is marked with a black dot (●), as determined by a neighbor-joining analysis. The source of the sequence was indicated after each protein name

The sequence analysis results showed that *SlWRKY28* contained a 1468-bp ORF, encoding 475 amino acids. The predicted protein isoelectric point was 7.62, average hydrophilicity was − 0.653, the aliphatic index was 62.22, stability index was 52.8, and the molecular weight was 52 kDa. The WRKY domain (Fig. 1a) contained a highly conserved motif with the sequence WRKYGQK and a C2H2 zinc finger motif fold at a position of 256–356 bp, which is a two-type WRKY TFs. Results showed that the homology of the NCBI amino acid BLAST was 92%. The putative WRKY clone from *S. linearistipularis*is was highly homologous to WRKY28 of *Populus davidiana* × *P. bolleana* (Fig. 1b) and it was designated as *SlWRKY28* (GenBank: MH223643).

### SlWRKY28 localizes to the nucleus

**Fig. 2:**
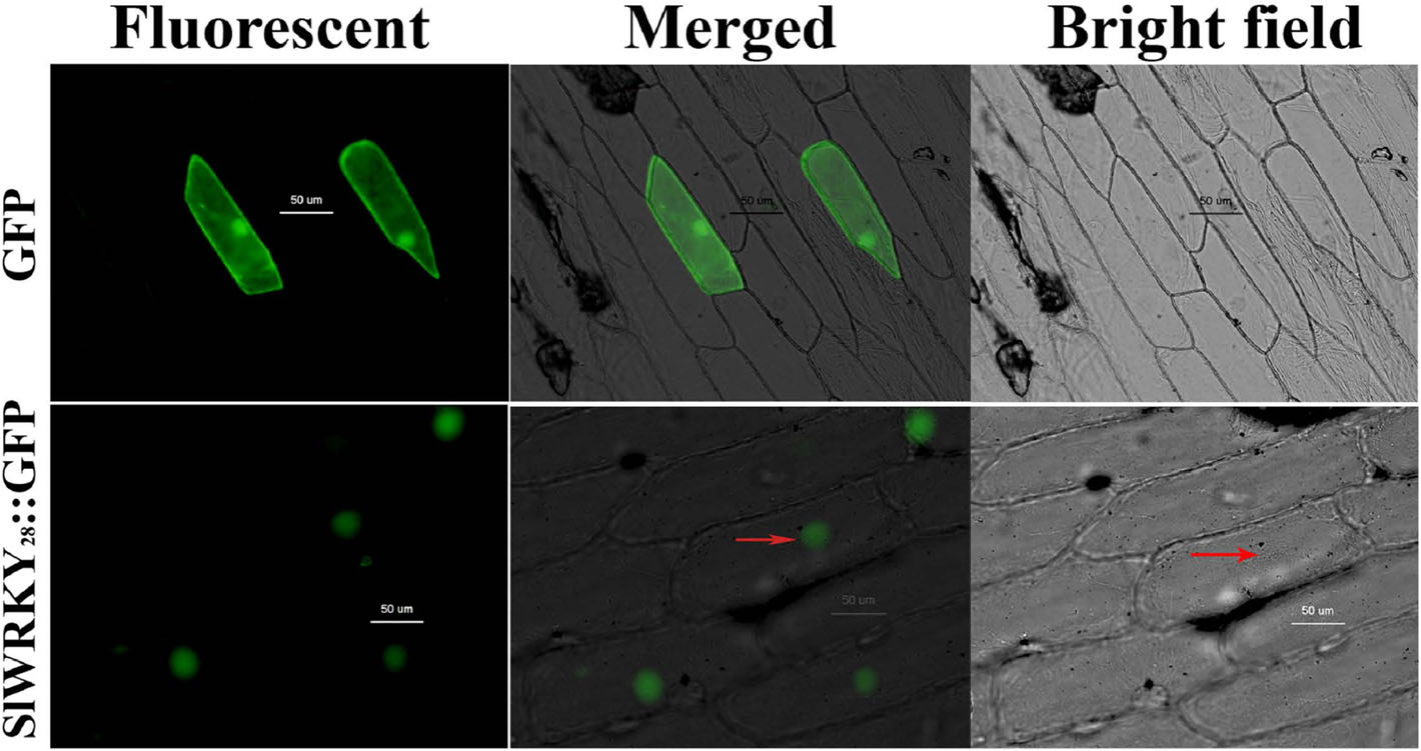
Subcellular localization of the 35S-SlWRKY28-GFP fusion protein. Merged, green fluorescence, and bright field superposition. (For interpretation of the references to color in this figure legend, the reader is referred to the web version of this article)

Consistent with the identified NLS sequence, the subcellular location prediction software Plant-mPloc (http://www.csbio.sjtu.edu.cn/bioinf/plant-multi/) predicted that the *SlWRKY28* protein localizes to the nucleus. To confirm our prediction, the 35S-*SlWRKY28*::GFP vector was constructed and transferred into onion epidermal cells. The 35S::GFP construct served as a control. The onion epidermal cells harboring the 35S-*SlWRKY28*::GFP construct emitted green fluorescence predominantly in nuclei (Fig. 2), whereas 35S::GFP fluorescence occurred widely throughout the cell [28].

### W-box DNA binding characteristics of SlWRKY28

**Fig. 3:**
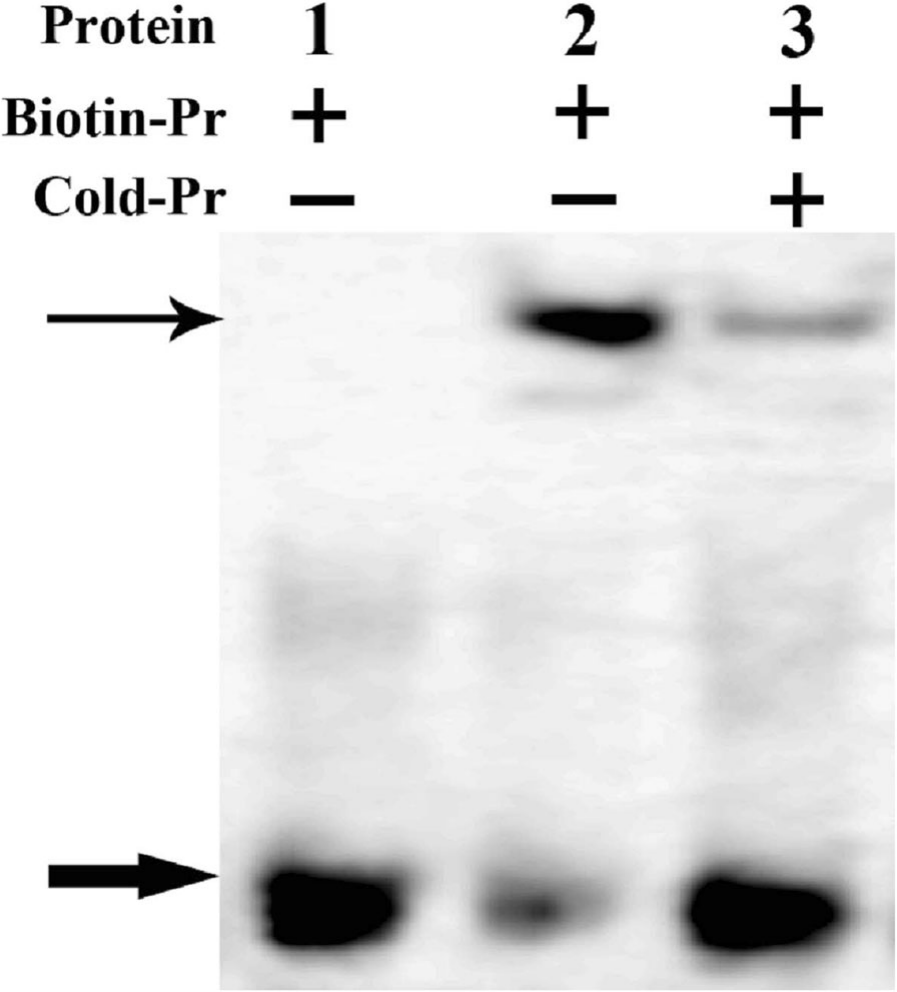
Combination of GST-SlWRKY28 fusion protein with W-box element. In the gel block, the lanes were followed by GST + protein and biotin-labeled oligonucleotide probe (Biotin-Pr); GST-SlWRKY28 fusion protein and Biotin-Pr were added; also undiluted oligonucleotide probe (Cold-Pr) diluted 10-fold; added GST-SlWRKY28 fusion protein and Biotin-Pr, and 50-fold diluted unlabeled oligonucleotide probe (Cold-Pr). The thin arrow refers to the DNA and protein complex; the thick arrow refers to the free probe

To detect the cloned *SlWRKY28* encoding protein can bind to DNA cis-element W-box with binding characteristic of WRKY TF, the prokaryotic expression vector *pGEX-6p-3-SlWRKY28* was constructed, and GST-SlWRKY28 fusion protein was expressed and purified using *E. coli* BL21 cells through IPTG induction. The gel blocking signal of GST-SlWRKY28 fusion protein and biotin-labeled probe were detected using the EMSA kit (Fig. 3). The swimming lane with only GST protein showed a free probe signal but no complex signal. This result indicated that the GST protein did not bind to the DNA probe. The complex of GST-SlWRKY28 fusion protein and Biotin-Pr produced the signal, whereas the complex signal was weak when a cold competitive probe was added. The fusion protein binds to the TTGAC amino acid of the DNA probe. The fusion protein could not bind to the cis-element W-box, indicating that the expressed SlWRKY28 could bind to a specific probe. The results revealed that the *SlWRKY28* encoding protein has the functional characteristics of the WRKY transcription factor binding to the W-box.

### Analysis of mRNA expression characteristics of *SlWRKY28*

**Fig. 4:**
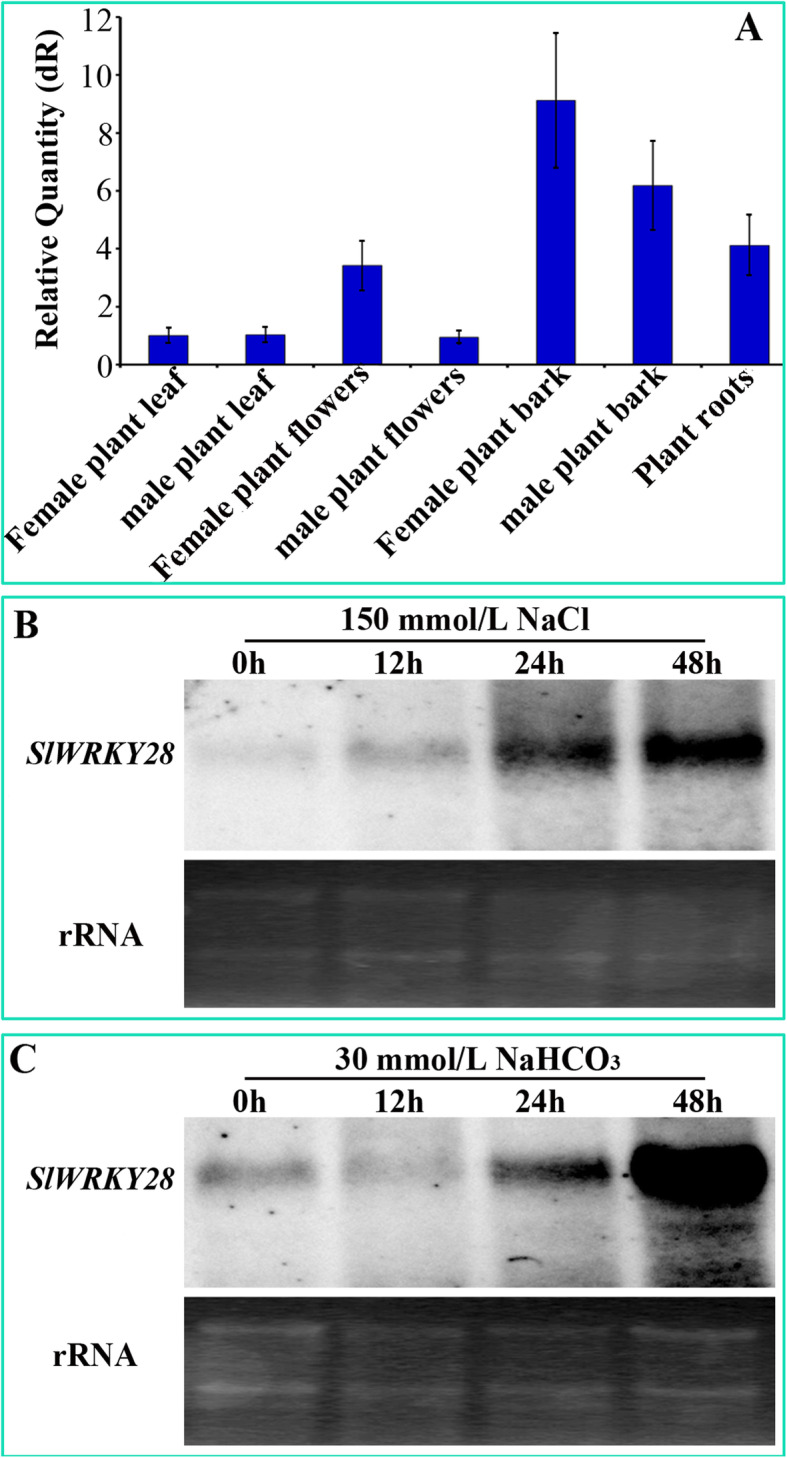
Different tissues, organs and under expression analysis of *SmWRKY28 gene.* **a** Specificity expression analysis of *SlWRKY28* in leaf flowers, plant bark, and roots of female and male plants. **b** Specificity expression analysis of *SlWRKY28* gene mRNA at different times of 150 mM NaCl and **c** 30 mM NaHCO_3_ stresses

The expression of *SlWRKY28* in the leaves, flowers, stem epidermis, anthers, and roots of male and female plants of the three-year-old *S. linearistipularis* was analyzed by qRT-PCR (Fig. 4a). Altogether, the expression level of female plants are higher than of male plants. The expression level of *SlWRKY28* increased with the prolongation of treatment time. Furthermore, expression of *SlWRKY28* was induced 12 h after 150 mM NaCl stress, and increased up to 48 h (Fig. 4b); in contrast, the response of *SlWRKY28* started 24 h after 30 mM NaHCO_3_, and increased significantly at 48 h (Fig. 4c). Those results indicated that *SlWRKY28* had a stronger response to NaHCO_3_ stress than to NaCl stress, and its response need a certain process.

### Expression analysis of *SlWRKY28* under NaHCO_3_ stress

**Fig. 5:**
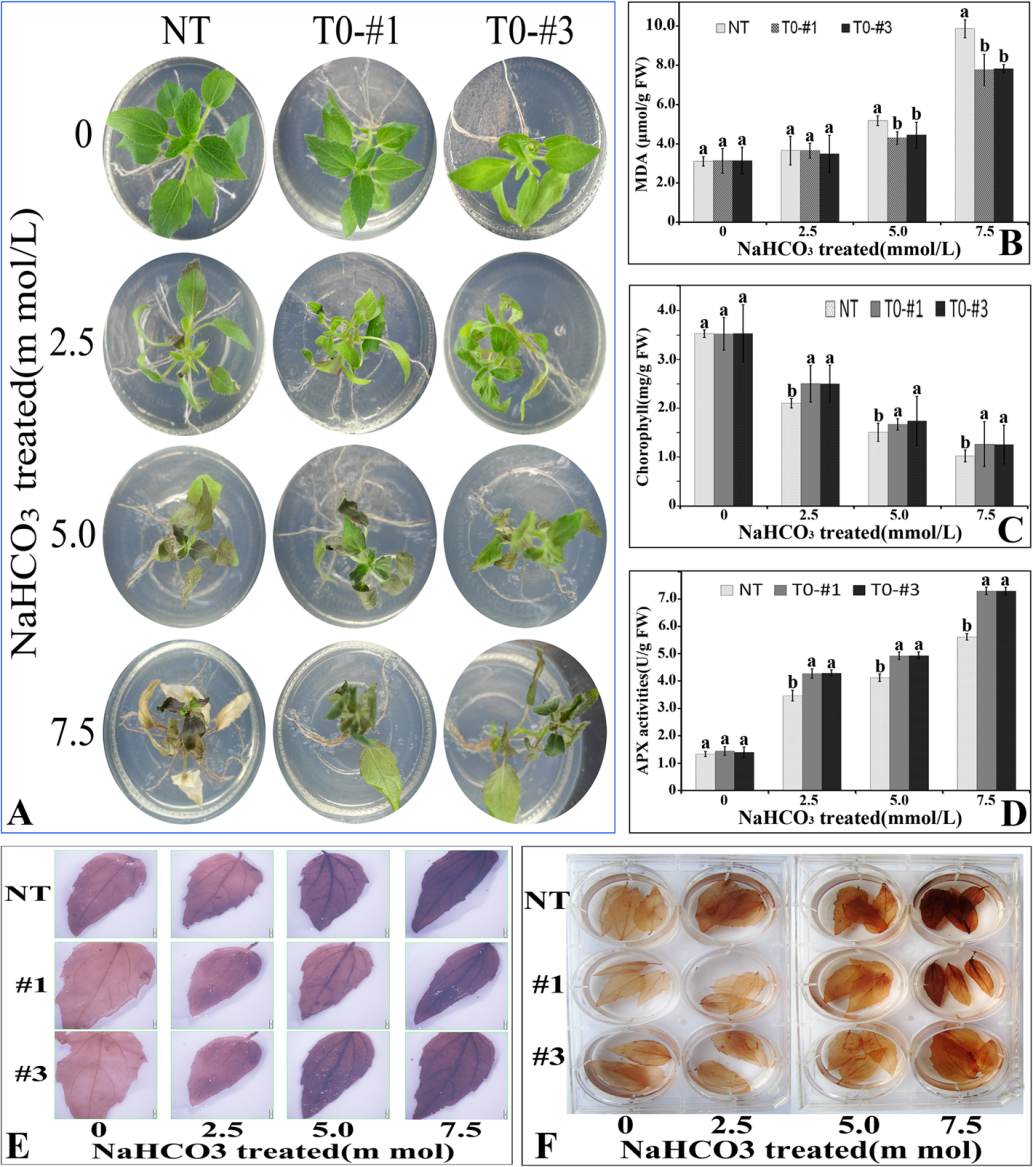
Tolerant growth of Populus overexpressing *SlWRKY28* under NaHCO_3_ stress. **a** Phenotypes of NT and overexpressed *SlWRKY28* in Populus (T0-#1,#3,#5) bottle seedlings under stress for 72 h. **b** MDA content. **c** Chlorophyll content. **d** APX enzyme Activity. **e,f** NBT and DAB staining results. Different lowercase letters indicate significant differences (*P* < 0.05) among different strains

The seedlings expressed *SlWRKY28* (T0 -#1,-#3) and NT was incubated on the 1/2 Murashige and Skoog (MS) medium with various concentrations of NaHCO_3_ (0, 2.5, 5, and 7.5 mmol/L), and phenotypes were investigated 72 h after the stresses (Fig. 5a). The phenotypes changed significantly under 5 and 7.5 mmol/L NaHCO_3_, and tolerance of overexpressing *SlWRKY28* plants to NaHCO_3_ was superior to NT. Under 5 and 7.5 mmol/L NaHCO_3_, malonic dialdehyde (MDA) concentration of overexpression *SlWRKY28* plants was significantly lower than NT (Fig. 5b), and the green content of leaves was higher than NT (Fig. 5c). Moreover, APX activity increased with the concentration of NaHCO_3_, and was significantly higher than NT plants (Fig. 5d).

The content of active oxygen species (O_2_ and H_2_O_2_) in *Populus davidiana* × *P. bolleana* treated with NaHCO_3_ (0, 2.5, 5, and 7.5 mmol/L) was expressed by nitro blue tetrazolium (NBT) and DAB. The results showed that the NT and transgenic plants deepened gradually with the increase of the stress concentration, the color of NT was darker than transgenic plants (Fig. 5e, f). These results showed that the content of O_2_ and H_2_O_2_ was lower than transgenic plants, which suggested the tolerance of *SlWRKY28* was increased to oxidative injury by NaHCO_3_ stress. Together, these results imply that SlWRKY28 TF may play an important role in gene expression regulation to anti-oxidative stress.

### Potential roles of *SlWRKY28* in regulating the expression of alkaline salt stress

**Fig. 6:**
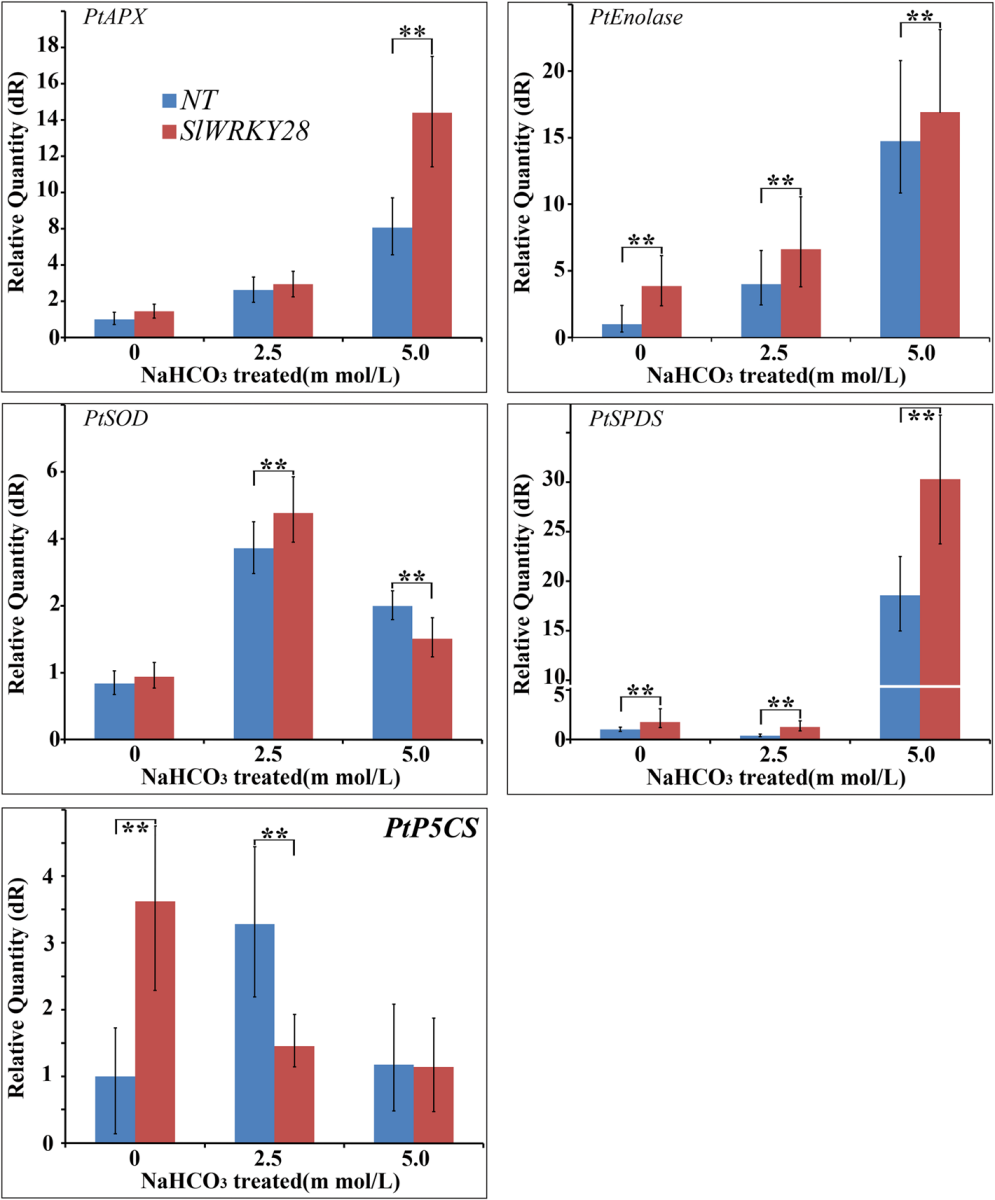
Expression of stress-related genes in *SlWRKY28* overexpressing and NT plants. Total RNA was extracted from *P. davidiana × P. bolleana* Loucne seedlings at the third-leaf stage grown under control and salt treatments. The transcript levels of *PtAPX, PtEnolase*, *PtSOD*, *PtSPDS*, and *PtP5CS* were measured using qRT-PCR under unstressed conditions of 0, 2.5 and 5.0 mmol/L NaHCO_3_ for 21 d, respectively. Data were written as mean and standard error of three replicates

In this study, *PtAPX, PtEnolase*, *PtSOD*, *PtSPDS*, *PtP5CS* genes in overexpressed lines T0-#1 and NT were further examined. These results show that the expression of PtAPX, PtEnolase and PtSPDS was increased of induced transcription, and the transgenic plants were significantly higher than those of NT plants. The transcription and expression levels of *PtSOD* increased under 2.5 mM NaHCO_3_, but when NaHCO_3_ concentration up to 5.0 mM, and the transcription expression of *PtP5CS* decreased rapidly under NaHCO_3_ stress. Those results suggest that SlWRKY28 TF may up-regulate the expression of *PtAPX, PtSOD, PtEnolase* and *PtSPDS* genes under the NaHCO_3_ stress, and down-regulate or not participate in the regulation of *PtP5CS* gene expression (Fig. 6).

### Analysis of photosynthetic characteristics of transgenic plants under NaHCO_3_ conditions

**Fig. 7:**
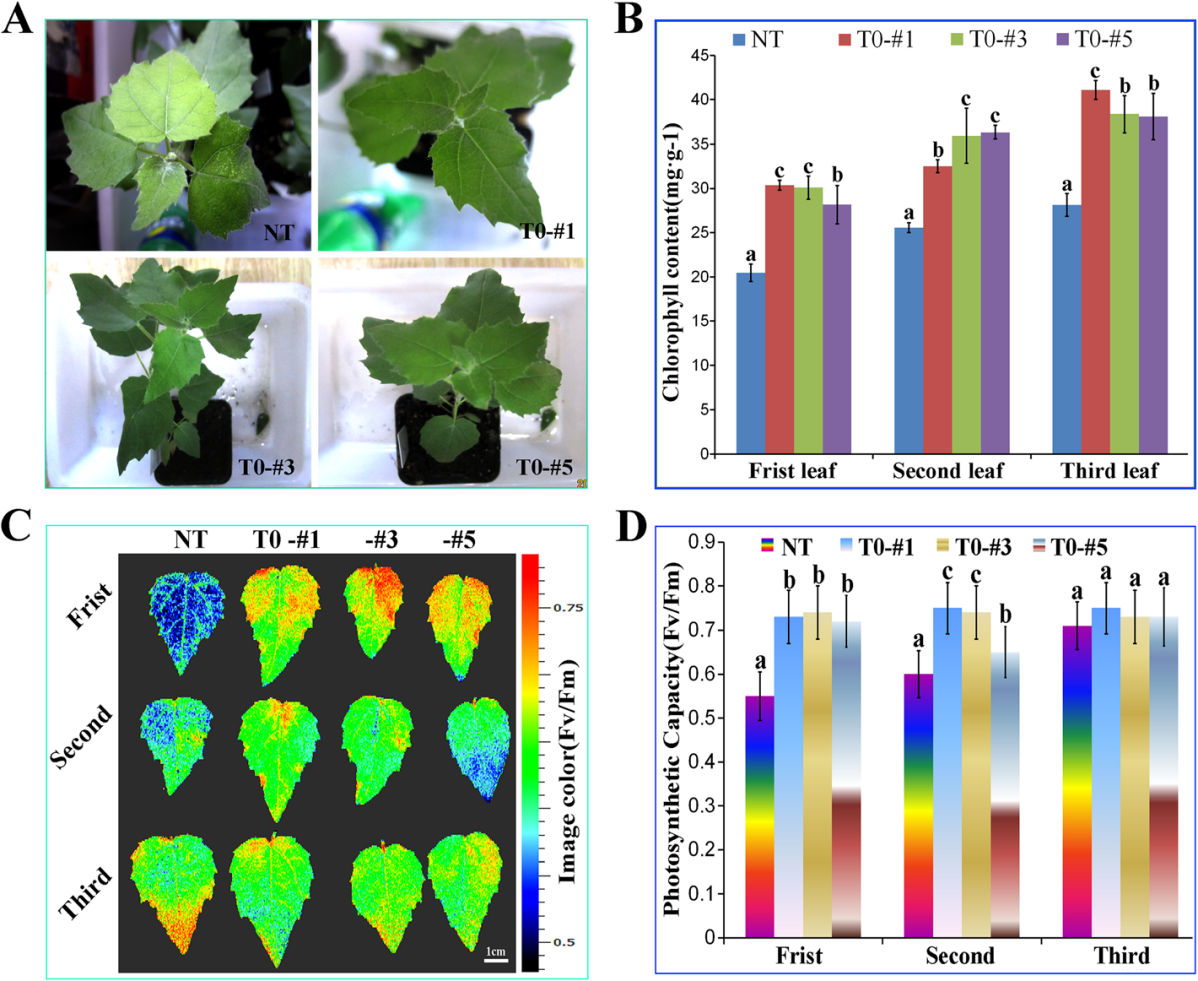
Photosynthetic capacity of overexpressed *SlWRKY28* and NT plants under NaHCO_3_ stress**. a** phenotype of the plant. **b** Chlorophyll concentration. **c** mapping imaging on the FluorCam open chlorophyll fluorescence system. **d** Comparison of photosynthetic capacity differences

Top leaves of NT plants turned yellow, whereas transgenic plants were bright green, (Fig. 7a) which have a significant difference intolerance of the parietal leaves to NaHCO_3_ stress. The chlorophyll concentration of transgenic plants was higher than NT (Fig. 7b). FluorCam chlorophyll fluorescence imaging system showed that the fluorescence intensity of NT gradually decreased from the third parietal leaf to the first one (Fig. 7c), indicate that photosynthetic capacity (Fv/Fm) has a downward trend. The photosynthetic capacity of transgenic plants was higher than NT, which indicates that overexpression of *SlWRKY28* can alleviate the damage caused by stress on photosynthesis and could improve tolerance of alkaline salt (Fig. 7d).

### Obtaining *SlWRKY28* transgenic plant

**Fig. 8:**
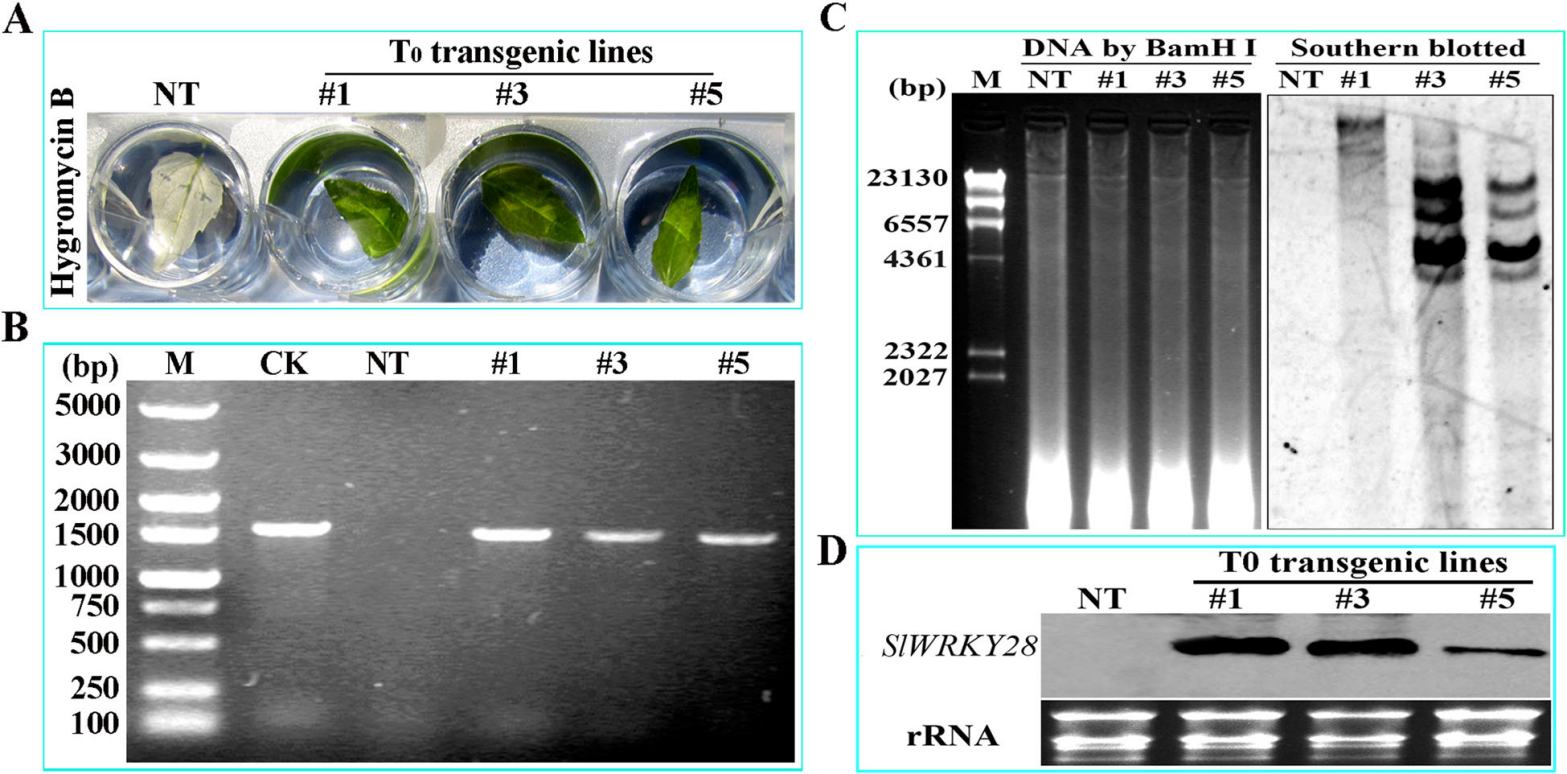
Identification of over-expression *SlWRKY28* gene plants(T0-#1,3,5 lines) in plants***.*** **a** Resistance screening of Hyg. **b** Detection picture of PCR**. c** Detection picture of Southern blotted. **d** Northern blot

The resistant transformants were obtained using *A. tumefaciens* to infect and transform leaves of *Populus davidiana* × *P. bolleana* in Hygromycin (Hyg) culture medium. The leaves of control soaked in Hyg 35 mg/L solution became white and the green recedes, but the transformed leaves (T_0_ -# 1, −# 3, −# 5) were still green (Fig. 8a). The targeted band via PCR indicated that *SlWRKY28* has been integrated into the chromosome of *Populus davidiana* × *P. bolleana* (Fig. 8b). Southern blot showed that T-#1 was a single copy insertion, T-#3, T-#5 lines were multi-copy insertions (Fig. 8c), strong hybridization signals were detected in T0-#1, −#3, −#5 lines and no signal in NT plant by Northern blot, those data confirmed SlWRKY28 expressed (Fig. 8d).

## Discussion

**Fig. 9:**
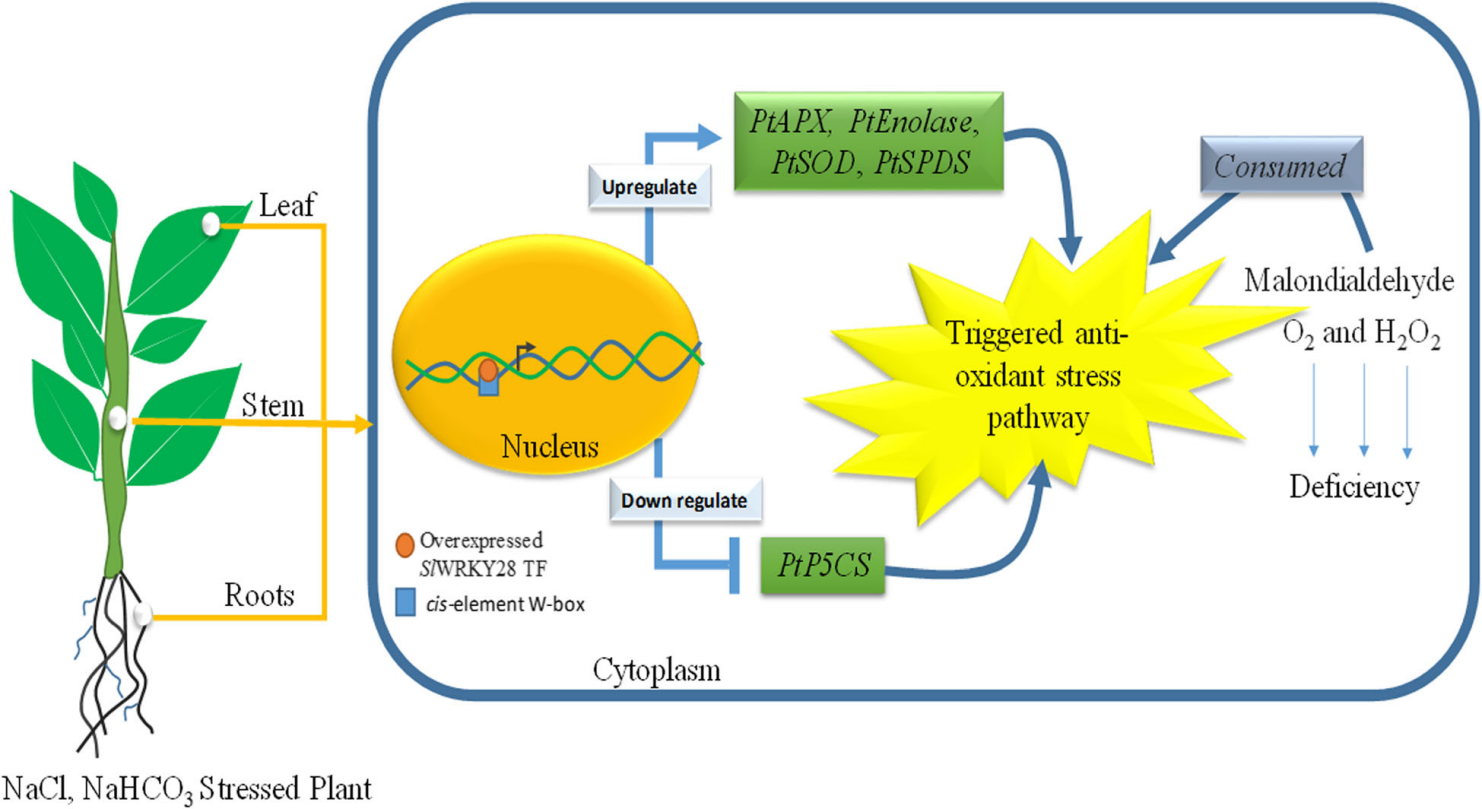
The salt tolerance mechanism in overexpressed SlWRKF28 TF under salt stress conditions

The WRKY TF is known as the largest superfamilies which participate in the regulation of proteins in plants [29]. In the past several years, growing evidence has shown that members of the WRKY gene family mainly participate in stress responses, plant growth and development and leaf senescence [30]. WRKY as an important TF can rapidly increase its family members to establish a signal transduction network in adversity, which may optimise plant adapt-ability [31]. In this study, we isolated a gene from *Salix linearistipularis* and characterized its functional roles. Many TFs families, such as WRKY, AP2 (APETALA2)/ERF (ethylene-responsive factor) and NAC (NAM, ATAF1/2, CUC1/2), are plant-specific transcription regulators [32]. zWRKY is not only involved in carbohydrate and secondary metabolite synthesis, senescence, and development but also plays an essential role in plant responses to biotic and abiotic stresses [33]. *S. linearistipularis* is an important salt and alkaline tolerant plant resource. In this study, the WRKY gene was cloned from *S.linearistipularis*, which encodes 475 amino acids with WRKY motif and a C2H2 zinc finger folding to form the WRKY domain (Fig. 1a). WRKY gene clone also accords with the characteristics of the IId and IIe subfamilies [34]. EMSA assay showed that encoding protein binds to TTAGC amino acids, and the binding characteristics of W-box *cis-*element also confirmed the function of WRKY TFs. The coding proteins of plant genes are based on the localization information contained in amino acid sequences specific parts mapping of the cell by precise guidance mechanism [35]. The PSORT analysis of biological software predicts that 73.9% of the SlWRKY28 protein is located in the nucleus; the verification experiment by gene gun bombarded the onion epidermal cells, which also confirmed that the fusion protein of 35S-SlWRKY28-GFP emitted green fluorescence in the nucleus (Fig. 2). The results of this study are consistent with the subcellular localization of WRKY TF studied using other species [36]. TFs and its structural gene jointly control growth and development traits, the WRKY TF may be involved in the regulation and responses of plants to drought and salt stress through pathways like other members of the WRKY TFs family [37, 38]. Northern blot analysis showed that the expression of *SlWRKY28* increased significantly with the prolongation of treatment time. Our findings indicate that *SlWRKY28* is a responsive gene to saline-alkali stress. The expression pattern was also found in *BCWRKY16*, *BCWRKY32*, and *BCWRKY35* in roots under NaCl and PEG6000 stress [39], as well as expression of *OsWRKY24, OsWRKY51, OsWRKY 71, and OsWRKY72* TFs under ABA, NaCl, PEG, cold and heat stress [40]. Various TFs are involved in modulating leaf senescence, and 1533 TFs have been identified via leaf senescence transcriptome analyses in *Arabidopsis* rice and cotton [41–43]. At present, studies on WRKYs in different species mainly focus on its roles in the signal transduction mechanism. In our study, *S. linearistipularis* has grown in a soda saline-alkali environment with a high pH value was selected. Our data showed that overexpression *SlWRKY28* was more resistant to NaHCO_3_ stress than NT plants, as indicated by higher green content of leaves and APX activity, with lower MDA concentration in the transgenic plants than NT. From the results, it can be inferred that *SlWRKY28* is potentially involved in the scavenging regulation of ROS and improvements in plant growth under alkaline salt stress. NBT and DAB staining showed that the color reaction in the third leaf was gradually deepened under NaHCO_3_ stress (Fig. 5e, f), and was lighter in the transgenic plants than in the NT plants. This result is consistent with changing patterns of POD, SOD, and Catalase (CAT) activities caused by overexpression of *DgWRKY3* in tobacco [44]. It is hypothesized that the *SlWRKY28* TF may be involved in the regulation of the expression of anti-oxidative stress -related genes. qRT-PCR analysis indicated that the expression of APX, Enolase and SPDS were increased under NaHCO_3_ treatment, with expression levels significantly higher in transgenic plants than in NT. Moreover, the expression of the SOD gene increased under 2.5 mM NaHCO_3_ stress but decreased when the stress increased to 5.0 mM NaHCO_3_. The transcriptional expression of the *P5CS* gene decreased rapidly under NaHCO_3_ stress, which suggests that *SlWRKY28* TF may up-regulate the expression of APX, SOD, enolase and SPDS genes under alkaline salt stress, and down-regulate or not contribute in the regulation of PtP5CS gene expression (Fig. 9).

These results suggest that *SlWRKY28* may play a significant role in the regulation of alkaline and salt stress tolerance. Moreover, *SlWRKY28* TFs may be involved in the regulation of photosynthetic electron transport pathway, which contributes to the improvement in plant tolerance to alkaline salts. The present study showed a theoretical and practical basis in potential pathways of *SlWRKY28* TFs in the regulation of plant tolerance to alkaline salt stress. However, further studies are needed to elucidate the pathways involved in the SlWRKY28 mediated mechanism.

## Conclusions

In this research work, we carried out the structure and functional characterization of the *SlWRKY28* in *Populus davidiana* × *P. bolleana*. *SlWRKY28* was involved in the regulation of alkali-resistant salt stress by regulating the downstream target genes associated with the ROS pathway. Our results provide a theoretic basis for investigating the functional mechanisms of exogenous genes in transgenic plants. However, other functions of the *SlWRKY28* gene require further study and clarification. Our work can further study the response of the over-expression of WRKY to the conditions of saline-alkali stress.

## Methods

### Plant materials and growth conditions

The seeds of *S. linearistipularis* were collected from Anda Experimental Base (46° 27′ N, 125° 22′ E) and stored in − 20 °C refrigerator at the Center for Research on Biological Resources and Environment of Saline-alkali Land, Northeast Forestry University, China. Leaves, stems (epidermis and xylem), flowers, and roots of three-year-old male and female *S. linearistipularis* plants were collected. The wild seedlings of *Populus davidiana* × *P. bolleana* were preserved at the Center for Research on Biological Resources and Environment of Saline-Alkali Land, Northeast Forestry University, China. *Escherichia coli* (*E.coli*) JM109 and *Agrobacterium tumefaciens* (*A.tumefaciens*) EHA105 were used for transformation experiments. Gene cloning vector pMD18-T, plant expression vector PBI121-MCS-GFP, and pGWB11 were purchased from Dalian Baobao Co., Ltd., China.

Plant RNA extraction kit (Beijing Kangwei Century Biotechnology Co., Ltd.), Blend Taq-Plus, and qRT-PCR fluorescent dye (2 × Brilliant III SYBR Green qPCR Master Mix) were purchased from TOYOBO Co., Ltd. GoTaq® Green Master Mix (Promega (Beijing) Biotechnology Co., Ltd.). Digoxigenin markers and CDP-Star chromogenic agents (Roche, Inc.), Electrophoretic mobility shift assay (EMSA) and BeyoECL kits (Biyuntian Biology Co., Ltd.), Cetyltrimethylammonium bromide (hexadecyl trimethyI ammonium bromide, CTAB; Harbin Nanchuan Biotechnology Co., Ltd.), hygromycin B (Hyg), rifampicin, and Carbapenem (Sigma Aldrich, USA) were purchased from the supplier. All analytical reagents of molecular grade or high purity were purchased from China and used in all experiments.

### Gene cloning and sequence analysis

To amplify the full-length cDNA and genomic sequences of SlWRKY28 primers were designed based on the coding sequence of SlWRKY28 submitted to NCBI. Under saline-alkali stress, the up-regulated expression of the *SlWRKY28* gene assessed based on RNA-Sequence in *S.linearistipularis*. *SlWRKY28* (*Sl28-F, R*) was cloned by RT-PCR. The protein domains [45], molecular weights, and isoelectric points and amino acid sequences of SlWRKY28 protein were analyzed by using Protparam Software. The subcellular localization of SlWRKY28 was predicted through PSORT, whereas the comparison of proteins sequence homology, online NCBI was used for the construction of the phylogenetic tree by Neighbor-Joining method in MAGA software.

### Subcellular localization of *SlWRKY28*

*pMD18-T-SlWRKY28* plasmid DNA was used as a template, and KpnI/SpeI site primers were added and inserted into T-vector via PCR and ligated into *pBI121-MCS-GFP* vector through enzyme digestion, which obtained *pBI121-SlWRKY28-GFP* plant localization expression vector. The green fluorescent expression of the *35S-SlWRKY28-GFP* fusion protein in the epidermal cells of the onion was detected under *Olympus* laser confocal microscope using a gene gun to bombard the onion bulb method and expression of subcellular localization was identified [46].

### Analysis of W-box binding characteristics of SlWRKY28 protein

The binding characteristics of the SlWRKY28 protein to W-box element was investigated with EMSA. The fusion protein (GST-SlWRKY28) was expressed in and purified from prokaryotic expression system as reported by Shan et al. (2014). The correct *pMD18-T-SlWRKY28* plasmid was digested with restriction endonuclease NotI and BamHI, and the target fragment was recovered. The *SlWRKY28* was constructed into the *pGEX-6p-3-SlWRKY28* expression vector with GST tag and digested using the same enzyme, and transformed into *E. coli* BL21 (DE3) cells. The expressed GST-SlWRKY28 fusion protein was purified through Glutathione Sepharose 4B (GE company) GST affinity chromatography. The purified fusion protein was used for gel block detection.

Preparation of oligonucleotide probe (Biotin-Pr): Probe primer for W-box was synthesize by Doctor Biology Co., Ltd., which were W-box F1: 5′-ggaacttgaccttgaccttagggctgcaggaattcg-3′; and reverse complementary sequence W-box R2: 5′-cgaattcctgcagccctaaggtcaaggtcaagttcc-3′. Each pair of primers for each probe combined two reverse complementary DNA sequences on a 1:1 scale which followed by denaturation at 100 °C for 10-min, synthesis of double-stranded DNA through gradually cooling to room temperature. An oligonucleotide probe (Biotin-Pr) was obtained by labeling two probes with biotin, and the unlabeled reverse complementary DNA was used as a cold competitive probe (Cold-Pr). According to the EMSA kit (Biyuntian Biological Co., Ltd.) instructions for EMSA slightly changed for optimizing conditions in our Laboratory.

Negative control reaction 0 consists of nuclease-free water, 7 μL; EMSA/gel-shift binding buffer, (5×) 2 μL; GST-SlWRKY28 fusion protein 0 μL; Biotin-Pr, 1 μL.

Sample reaction 1 includes nuclease-free water, 5 μL; EMSA/gel-shift binding buffer (5×) 2 μL; GST-SlWRKY28 fusion protein 2 μL; Biotin-Pr 1 μL.

Probe cold competition reaction 2 has mixture of nuclease-free water, 4 μL; EMSA/gel-shift binding buffer, (5×) 2 μL; GST-SlWRKY28 fusion protein, 2 μL; Biotin-Pr, 1 μL; 10 × Cold-Pr, 1 μL.

Probe cold competition reaction 3 was performed with nuclease-free water 4 μL; EMSA/gel-shift binding buffer (5×) 2 μL; GST-SlWRKY28 fusion protein 2 μL; Biotin-Pr 1 μL; 50 × Cold-Pr 1 μL.

Mix properly and react at room temperature 25 °C for 20 min, 1 μL EMSA/gel-shift sample buffer was added for SDS-PAGE gel electrophoresis and the membrane was transferred. Purple diplomatic union (UV-light cross-linker 254 nm), BeyoECL moon A and B solution (Biyuntian Biological Co., Ltd.) was used to mix for color and combined with imaging detection on LAV4000.

### Analysis of mRNA expression characteristics of *SlWRKY28*

For analysis of expression characteristics of tissues and organs, male and female flowers, terminal buds, tuck buds, leaves, and stem segments (epidermis and xylem) were collected from three-year-old *S. linearistipularis.* RNA was extracted from the roots of the plant and rverse-transcribed into cDNA. The *Actin1* gene was used as an internal reference to normalize the gene expression of all samples. The control group was set with plain water. The data acquisition was completed on the MxPro-Mx3000P system, and each response was repeated three times. At the end of the reaction, the relative expression of the gene was calculated using relative quantitative method. The output was analyzed in Excel format, and a column graph was constructed according to the change of expression quantity.

Analysis of expression characteristics after stress treatment. Extraction of total RNA from *S. linearistipularis* under NaCl and NaHCO_3_ using RNA Kit. Agarose (1.2%) denatured gel electrophoresis (15 V/cm) was run for 4 h, then transferred RNA on membrane. *SlWRKY28* specific probe (qRT-F/R 496 bp) labeled with DIG was hybridized and then washing the membrane. The CDP-Star color-developing agent was used to detect signals on the Image Quant LAS 4000 imaging analyzer [47].

### Growth tolerance of overexpressed *SlWRKY28* seedlings to NaHCO_3_ stress

The growth-consistent overexpressing plants of *SlWRKY28* and NT (T0 / 1, # 3 lines) were inoculated into 1/2 MS medium containing 0, 2.5, 5, and 7.5 mM NaHCO_3_ (three replications per treatment). Plants were grown at 22 °C room temperature, 16/8 h light/dark period, and growth parameters of the plants were investigated under stress. Concentrations of chlorophyll and MDA in the third parietal leaf and the activity of APX in the whole plant were measured.

### Detection of photosynthesis ability of overexpressed *SlWRKY28* under NaHCO_3_ stress

Transgenic plants were cultured in a pot of 5 cm × 5 cm for 1 month under 600 μmol m^− 2^ s^− 1^ light intensity. Selecting healthy NT and overexpressing *SlWRKY28* plants, the phenotype was observed for 7 days with a 0.1 mol/ L NaHCO_3_ solution (3 replicates). The content of chlorophyll and the ability of photosynthesis Fv/Fm in the third parietal leaf were detected by FluorCam open chlorophyll fluorescence imaging system [48].

### Vector construction and plant transformation

**Fig. 10:**
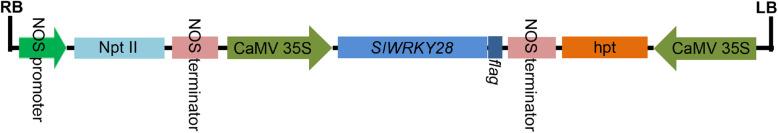
A flow chart of plant expression vector of *SlWRKY28* construction (*pGWB11-SlWRKY28*)

The binary plant expression vector *pGWB11-SlWRKY28* (Fig. 10) was connected, through the gateway system and electro-transformation into *A.tumefaciens* EHA105. Genomic DNA from pot-transformed plants was extracted through CTAB. The target gene was aligned for PCR detection. The inserted copy number in this generation strains was detected by Southern blot [49]. *SlWRKY28* expression in transgenic plants was detected by northern blot [47].

### Statistical analyses

Microsoft Excel 2010 (Microsoft Corp., Redmond, WA) and Statistical Product and Service Solutions v. 20.0 (SPSS, Chicago, IL) were used to analyze the experimental data. Both one-way analyses of variance and two-way analysis of variance were used to determine the significance of the differences among treatments. Student’s t-test was run to calculate *P*-values (*P < 0.05; **P < 0.01). The data were normalized, and all samples were normally distributed in terms of homogeneity of variance.

## Data Availability

The datasets generated and analysed during the current study are available in the [GenBank: MH223643] https://www.ncbi.nlm.nih.gov/ repository.

## Abbreviations

APX: Ascorbate peroxidase
ABA: Abscisic acid
CAT: Catalase
DAB: 3,3′-Diaminobenzidine
H_2_O_2_: Hydrogen peroxide
Hyg: Hygromycin B
JA: Jasmonic acid
MS: Murashige and Skoog
MDA: Malonic dialdehyde
NT: Non-transgenic
NBT: Nitro blue tetrazolium
ROS: Reactive oxygen species
SA: Salicylic acid
TFs: Transcription factors
